# Supplementary material for: Genomic and Transcriptomic Dissection of Growth Characteristics and Exopolysaccharide-Related Bioactivities in Lactiplantibacillus plantarum NMGL2
Source: Foods. 2025 Oct 16;14(20):3520. doi: 10.3390/foods14203520 (PMC12564313; doi:10.3390/foods14203520)
Supplement: Supplementary file 1 [file foods-14-03520-s001.zip › Supplementary Figure S1.pdf]

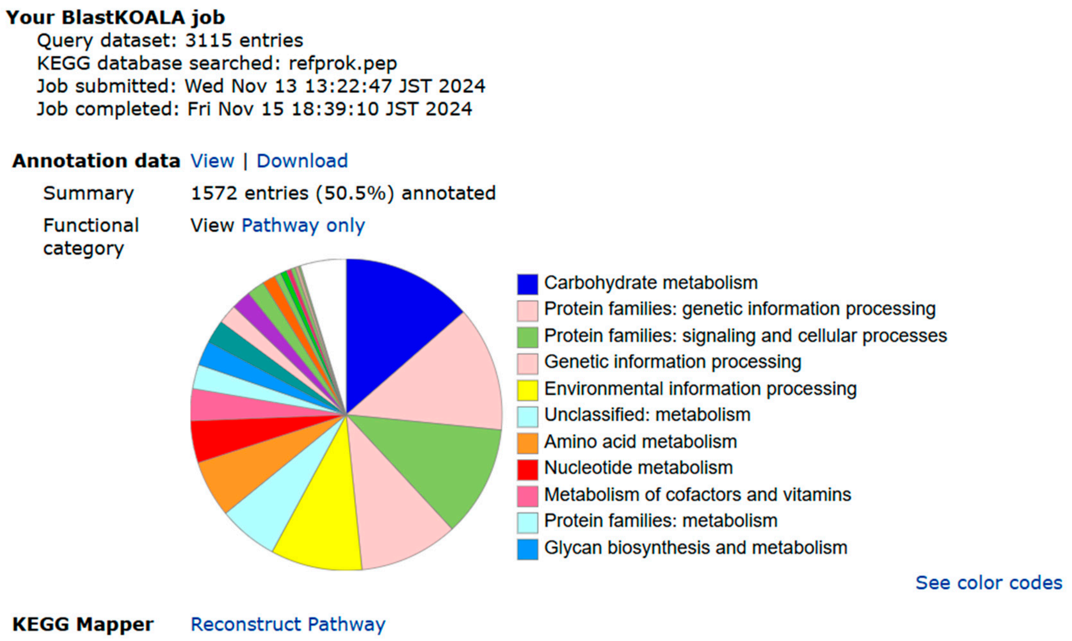

Figure S1. BlastKOALA annotation functional category summary
